# The effects of different exercise interventions on reducing internet addiction in adolescents or young adults: a systematic review and network meta-analysis

**DOI:** 10.3389/fpsyt.2025.1713076

**Published:** 2025-11-26

**Authors:** Ying Li, Songwei Zhong, Li Lian

**Affiliations:** 1 College of Sports Science, Jishou University, Jishou, Hunan, China; 2 School of Physical & Health Sciences, Guangxi Science & Technology Normal University, Laibin, Guangxi, China; 3 Physical Education Department, Beijing Wuzi University, Beijing, China

**Keywords:** internet addiction, teenagers, network meta-analysis, IA, adolescents

## Abstract

**Background:**

Adolescents or young adults’ physical and mental health, along with their academic performance, are negatively impacted by Internet addiction (IA), with such behavior being associated with the onset of cognitive and mental health disorders. Consequently, this issue has emerged as a pressing global social problem that demands urgent resolution.

**Objective:**

This research employed a meta-analytic approach to systematically assess the efficacy of diverse exercise-based interventions in mitigating IA among university students. The primary objective was to determine optimal therapeutic exercise modalities and formulate evidence-based guidelines for subsequent intervention strategies targeting adolescent internet overuse.

**Methods:**

A comprehensive systematic literature search was conducted across multiple international and domestic databases, including Web of Science, PubMed, Embase, Cochrane Library, China Knowledge, and Wanfang. Methodological quality was evaluated utilizing the revised Cochrane Risk of Bias tool for randomized trials. Subsequently, both conventional and network meta-analyses were performed employing Review Manager 5.3 and Stata 14.0 statistical software packages.

**Results:**

Traditional meta-results showed that exercise intervention was better than the control group in improving adolescent IA (SMD= -2.33, 95%CI -3.00, -1.66). Network meta-analysis(NMA) showed that Combined movement (CM) improved adolescent IA better than Control group (CG) (SMD-3.47, 95% -4.85, -2.10), and CM had the highest probability of being the best intervention for IA (SUCRA = 86.7%).

**Conclusion:**

Exercise-based interventions demonstrate significant therapeutic efficacy in addressing IA, with CM exhibiting superior effectiveness for adolescent populations. Nevertheless, given the methodological limitations imposed by restricted sample sizes and heterogeneous literature quality, future large-scale randomized controlled trials are warranted to validate these preliminary findings.

**Systematic review registration:**

https://www.crd.york.ac.uk/prospero/, identifier CRD420251006694.

## 1 Introduction

The Statistical Report on China’s Internet Development (2022) reveals a national internet user base of 1.067 billion by December 2022, reflecting a year-on-year growth of 35.49 million users from 2021 figures. This expansion corresponds to an internet penetration rate of 75.6% across the country’s population (1). While the Internet significantly enhances efficiency in both daily life and professional domains, excessive reliance on digital connectivity may precipitate the onset of Internet Addiction (IA). IA, clinically recognized as a behavioral addiction, manifests through compulsive online engagement and impaired impulse control independent of substance influence (2). This condition demonstrates core addictive characteristics, including compulsive use patterns, diminished self-regulation, and withdrawal difficulties. Furthermore, IA adversely affects academic performance while increasing risks of delinquency and self-harm, necessitating urgent multidisciplinary intervention from families, educators, and policymakers (3).

Therapeutic approaches for IA primarily encompass psychological interventions, pharmacological treatments, and clinical management. However, affected adolescents or young adults frequently exhibit treatment non-compliance due to social stigma and psychological reactance, often resulting in premature discontinuation. This treatment attrition stems from inherent limitations of conventional modalities, including protracted duration, substantial financial burdens, and significant adverse effects (4). It has been found that sports can effectively reduce IA behavior by repairing structural brain damage, improving brain functional connectivity, and bi-directionally modulating dopamine and its receptors (5). Physical exercise, recognized for its low cost, widespread popularity, and high compliance, has been demonstrated to be a highly effective and significant intervention in the treatment of both substance and behavioral addiction. Gao (6) and Zhang (7) found in their research that exercise intervention can effectively improve IA in teenagers. This study demonstrates that competitive team sports, such as football and basketball, through their inherently engaging and enjoyable nature, effectively improve feelings of accomplishment, social belonging, and interpersonal competence in adolescents or young adults with IA while simultaneously mitigating the severity of addiction (8, 9). Zhang (10) et al.’s 12-week physical exercise intervention (jogging, basketball, and outdoor training) significantly reduced IA levels among college students. Zhang (11) found in his research that Tai Chi can significantly reduce the IA of college students. In the study, Xiao (12) discovered that a short-term basketball intervention yielded superior results compared to the Baduanjin intervention in alleviating symptoms and enhancing the mental well-being of mobile phone addicts. Despite direct comparisons being made between Tai Chi (11), basketball (10), and Baduanjin (10), indirect comparisons among specific sports are still lacking. Therefore, the most effective sports for addressing IA in teenagers remain unclear.

Network meta-analysis (NMA) has gained prominence in evaluating medical interventions due to its capacity to estimate the relative effectiveness and ranking of interventions, even in the absence of direct comparisons (13). In a NMA of 11 interventions, Zhou (14) and Zhang (15) found that exercise may be the most appropriate single intervention for reducing adolescent IA. However, it remained unclear which type of exercise intervention was most effective. Therefore, this study examined the effects of exercise interventions on adolescent IA, incorporating a detailed classification of exercise modalities, to identify the optimal intervention for reducing IA in this population. This research aims to guide adolescents or young adults with IA in choosing the most effective exercise regimen, reduce severe IA among youths, and promote healthier lifestyles.

## 2 Methods

This study was reported per the PRISMA NMA guidelines (16). The review protocol was registered with the International Prospective Register of Systematic Review (PROSPERO) (CRD420251006694).

### 2.1 Search strategy

The computer searched PubMed, Web of Science, Embase, Cochrane Library, CNKI, and other databases, and the search period was established until February 22, 2025. The search takes the way of combining subject words and free words. The search strategy uses Pubmed as an example, as shown in **Appendix 1**.

### 2.2 Study selection

The inclusion criteria for study selection were based on the PICOS methodology (Participants, interventions, comparators, outcomes, and study design) (16), shown in **Table 1**.

**Table 1:** Inclusion and exclusion criteria.

Category	Inclusion criteria	Exclusion criteria
Population	A clear diagnosis of IA in Adolescents and Young Adults (10-24Years) (17)	Adolescents or young adults with IA who receive cognitive behavioral therapy or drug therapy Participants with anxiety and depression
Interventions	Tai Chi (TC), Baduanjin (BDJ), Other exercise (OE), Running exercise (RE), Cycling exercise (CE), Combined movement (CM), Basketball exercise (BE), Football exercise (FE). a) a frequency of at least two sessions per week; b) a total duration of no less than six weeks; and c) exercise intensity prescribed as, or equivalent to, moderate intensity.	
Comparisons	Control group (CG)	
Outcomes	Using Smartphone Addiction Scale Shortened Version (SAS-SV, >32 scores), Young’s IA Scale (YIAS, >5 scores), Chen’s Chinese IA Scale (CIAS-R, >68 scores) and Mobile Phone Addiction Index (MPAI, >34 score) were diagnosed as IA. These scales have excellent reliability and validity (18, 19).	
Study	Randomized controlled trial; published in English or Chinese	duplicate publications; conference papers and review articles.

CM: Two or more of the specific types of exercise training (not deemed multimodal if only part of warm up or cool down) (20).

### 2.2 Data extraction

Two independent reviewers extracted the following data: first author, publication year, country of origin, sample size, intervention method, duration of intervention, and intervention period. The data are presented as mean ± standard deviation (mean ± SD). In cases where multiple time points for the outcome measures are reported, data from the most recent time point were extracted.

### 2.3 Risk of bias assessment

The risk of bias was independently evaluated by two reviewers, with a third reviewer involved in the assessment, utilizing the tools recommended by the Cochrane Collaboration (21). These tools included criteria such as sequence generation, concealment of allocation, blinding, incomplete outcome data, selective reporting of results, and other potential sources of bias. Each criterion was classified as having a low, unclear, or high risk of bias.

### 2.4 Data analysis

Meta-analysis was conducted using RevMan 5.3 software. The mean difference (MD) was employed as the effect size for continuous data, and the effect sizes of both the experimental and control groups were pooled using a random-effects model. A 95% confidence interval (95% CI) was provided for each effect size. The heterogeneity of effect sizes across studies was quantitatively assessed using the I² statistic. A threshold of I² > 50% or a p-value ≤ 0.10 for the Q test was considered indicative of significant heterogeneity (21). In the presence of substantial heterogeneity, a random-effects model was applied, whereas a fixed-effects model was used when heterogeneity was minimal. Subgroup analyses were performed based on the characteristics of the interventions and populations. Sensitivity analysis was conducted to identify the source of heterogeneity in studies exhibiting substantial variability, testing whether any individual study contributed significantly to the observed heterogeneity.

NMA was also conducted to perform a random-effects multivariate NMA for pooled estimates within the frequentist framework (22). The network structure was visually represented in a network plot, where the connections between nodes indicated direct head-to-head comparisons of interventions. The size of each node and the thickness of the lines connecting them were proportional to the number of studies included. A network contribution graph was generated to assess the contribution of each direct comparison. To examine discrepancies between direct and indirect comparisons, loop-specific heterogeneity estimates, an inconsistency model, and node-splitting analysis were applied. The surface under the cumulative ranking curve (SUCRA) was utilized to rank and compare the effectiveness of different interventions. SUCRA values range from 0 to 100, with a value of 100 signifying the optimal treatment with no uncertainty, and a value of 0 indicating the least effective treatment without uncertainty (23). Moreover, a network funnel plot was generated to check for publication bias.

## 3 Results

### 3.1 Study selection

**Figure 1:**
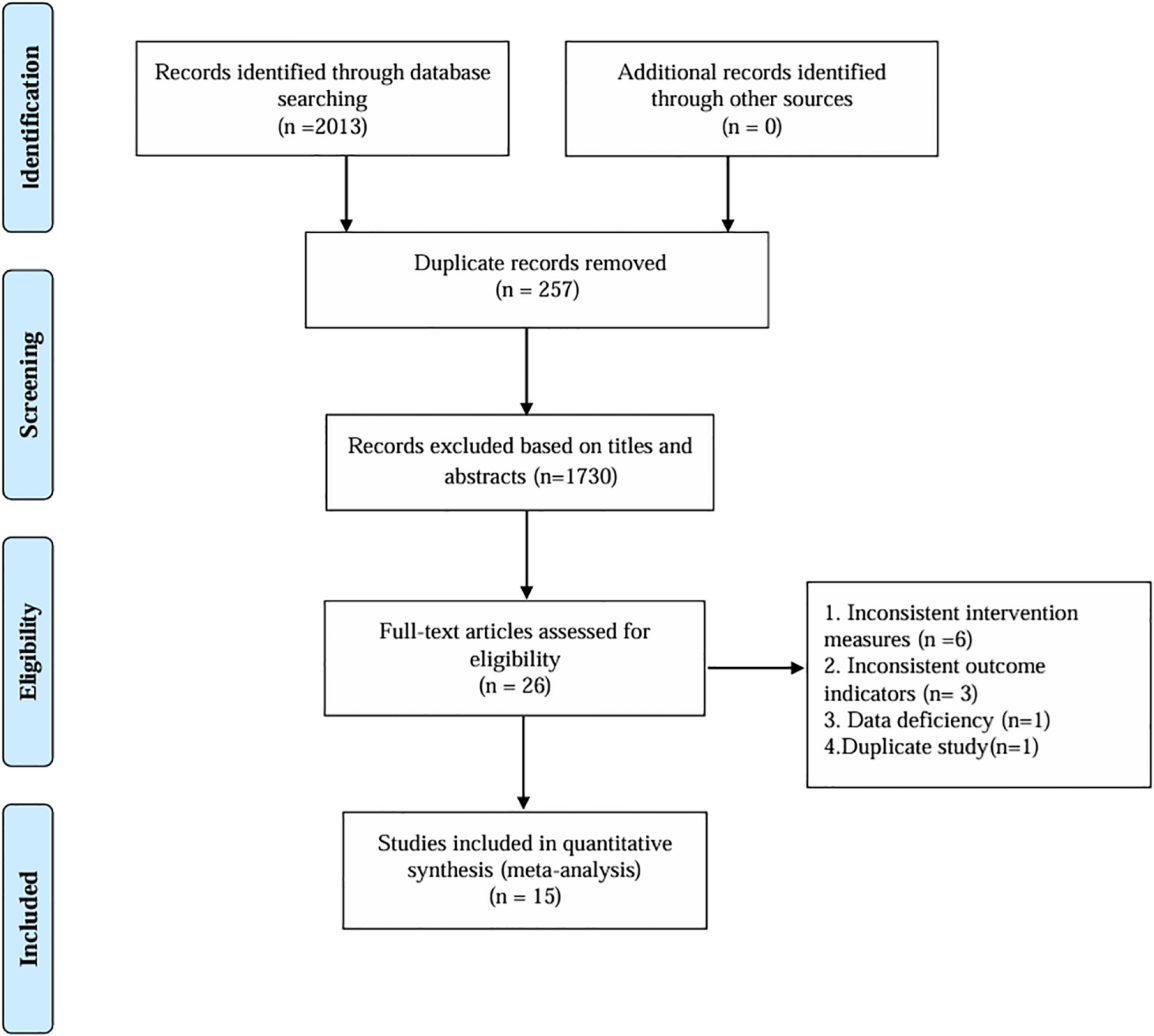
PRISM flow diagram.

After deleting duplicates, 2013 records were retrieved, 257 duplicates were removed, 1730 articles with inconsistent titles were deleted (This process was carried out by two independent reviewers who used the Endnote software. Any differences among the reviewers shall be resolved through discussion. If necessary, they may be negotiated with a third senior researcher), 11 articles with inconsistent titles were removed after reading the full text, and 15 articles were finally included. The research flow chart is shown in **Figure 1**.

### 3.2 Basic information included in the study

As shown in **Table 2**, the 15 included studies included 920 adolescents or young adults with IA. For outcome measures, five studies used CIAS-R to assess the severity of IA; eight studies used YIAS, with one item each for MPAI and SAS-SV. Ten studies were published before 2020, and five were published after 2020.

**Table 2:** Detailed characteristics of the included studies.

Author	Publish year	Country	Measure	Age (mean ± SD)	Number	Intervention time	Intervention frequency	Exercise facility	Exercise intensity	Outcomes
Xiao (12)	2021	China	BDJ/BE/CG	BDJ=19.21 ± 1.02 BE=18.95 ± 0.89 CG = 19.71 ± 1.77	31/31/34	12 Weeks	3 times/Week	School	Moderate-intensity exercise	MPAI
Zhang (25)	2024	China	CM/TC/CG	CM=20.03 ± 0.556 TC=20.10 ± 0.759 CG=20.20 ± 0.610	30/30/30	8 Weeks	3 times/Week	School	Moderate-intensity exercise	SAS-SV
Zhang (26)	2009	China	CM/OE	NA	35/35	12 Weeks	2 times/Week	School	Moderate-intensity exercise	CIAS-R
Gao (27)	2012	China	CE/CG	NA	35/34	8 Weeks	5 times/Week	School	Moderate-intensity exercise	YIAS
Xie (28)	2022	China	CM/CG	NA	40/40	16 Weeks	3 times/Week	School	Moderate-intensity exercise	YIAS
Yang (29)	2017	China	TC/CG	TC=19.6 ± 1.2 CG=19.7 ± 1.4	26/26	16 Weeks	4 times/Week	School	Moderate-intensity exercise	CIAS - R
Zhang (30)	2013	China	CM/CG	NA	30/30	16 Weeks	2 times/Week	School	Moderate-intensity exercise	YIAS
Zhu (31)	2008	China	BE/CG	NA	6/6	12 Weeks	3–4 times/Week	School	Moderate-intensity exercise	YIAS
Fu (8)	2016	China	FE/CG	NA	42/42	16 Weeks	3 times/Week	School	Moderate-intensity exercise	YIAS
Yang (32)	2021	China	BE/RE/CG	NA	28/28/28	12 Weeks	2–3 times/Week	School	Moderate-intensity exercise	CIAS - R
Deng (33)	2014	China	CM/CG	NA	24/24	10 Weeks	3 times/Week	School	Moderate-intensity exercise	CIAS - R
Ren (34)	2014	China	CM/CG	NA	4/4	12 Weeks	3 times/Week	School	Moderate-intensity exercise	YIAS
Wen (35)	2020	China	RE/CG	RE=20.63 ± 2. 06 CG = 20.34 ± 1.24	40/40	8 Weeks	3–4 times/Week	School	Moderate-intensity exercise	CIAS - R
Li (36)	2014	China	CM/CG	CM=15.41 ± 1.47 CG=15.62 ± 1.78	27/24	10 Weeks	3 times/Week	School	Moderate-intensity exercise	YIAS
Qiu (37)	2011	China	CM/CG	NA	18/18	12 Weeks	3 times/Week	School	Moderate-intensity exercise	YIAS

### 3.3 Methodological quality assessment

**Figure 2:**
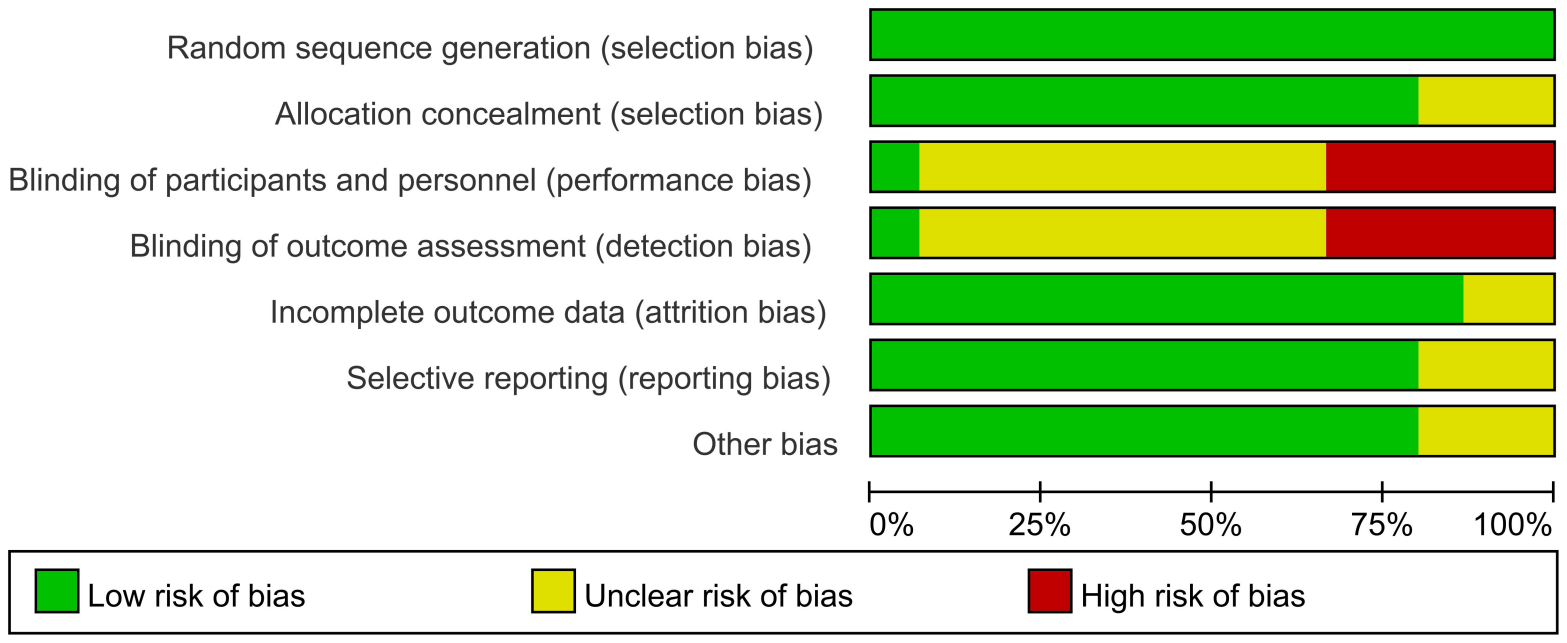
Summary of risk of bias.

The methodological quality of the 15 included articles was assessed. Studies with no high risk and only three or fewer studies rated as ambiguous risk were classified as low risk. There was one high-risk point or five high-risk points, but four or more were rated as risky and not explicitly classified as medium-risk points. All the other conditions were classified as high-risk points (24). The summary of the bias risk assessment is shown in **Figure 2**.

### 3.4 Meta-analysis

**Figure 3:**
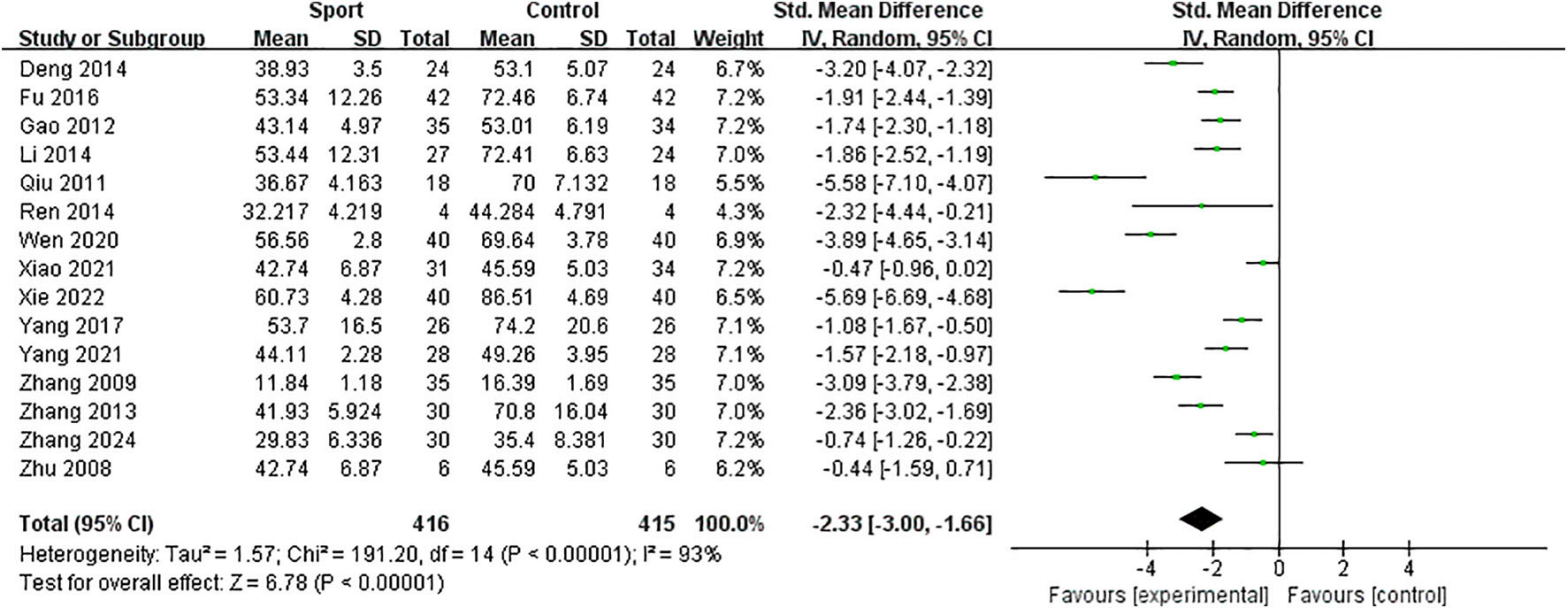
Impact of interventions on IA.

Effect of exercise measures compared with the control group. A meta-analysis of 15 studies was performed. The overall results are shown in **Figure 3**. Compared with the control group, the exercise intervention significantly reduced adolescent IA [SMD= -2.33, 95%CI (-3.00, -1.66), p < 0.001], and *I^2^* showed significant heterogeneity (*I^2^* = 93%, p < 0.001).

#### 3.4.1 Subgroup analysis

To further investigate the sources of heterogeneity, we performed a meta-regression using Stata 15 (**Appendix 4**). Subsequently, sensitivity analyses were conducted using RevMan 5.3; however, the heterogeneity (*I*²) remained above 50% even after applying the leave-one-out method. We therefore proceeded with a series of subgroup analyses based on sample size, exercise type, outcome measurement, intervention duration, publication year, and exercise frequency. The results of these analyses are presented in **Table 3**.

**Table 3:** Subgroup analysis to assess the effect of interventions on adolescents or young adults ‘ intervention addiction.

Variable	Number of trials	Sample size		Meta-analysis			Heterogeneity		
		EG	CG	SMD	CI	P^a^	I^2^	Chi^2^	P^b^
All	15	416	415	-2.33	-3.00, -1.66	—	98%	191.20	<0.001
Year of publication									
Before 2020	10	247	243	-2.26	-2.87, -1.65	0.84	84%	56.51	<0.001
After 2020	5	169	172	-2.43	-4.04, -0.82		97%	129.17	<0.001
Sample size									
<50	4	52	52	-2.87	-4.95, -0.80	0.53	90%	30.09	<0.001
≥50	11	364	363	-2.17	-2.90, -1.45		93%	152.40	<0.001
Outcome measurement									
CIAS-R	5	153	153	-2.55	-3.62, -1.47	<0.001	92%	48.01	<0.001
YIAS	8	202	198	-2.68	-3.65, -1.70		91%	79.30	<0.001
Other	2	61	64	-0.60	-0.96, -0.24		0%	0.54	0.46
Intervention duration									
8–10 Weeks	4	126	122	-2.65	-3.68, -1.62	0.54	88%	25.96	<0.001
12 Weeks	7	152	155	-1.91	-2.94, -0.88		92%	74.43	<0.001
16 Weeks	4	138	138	-2.71	-3.00, -1.66		95%	61.59	<0.001
Intervention frequency									
2 times/week	2	65	65	-2.71	-3.43, -2.00	0.59	54%	2.17	<0.001
3 times/week	9	222	222	-2.40	-3.47, -1.33		94%	139.45	<0.001
other	4	129	128	-2.05	-3.12, -0.98		92%	35.47	<0.001
Intervention mode									
Other exercises	10	285	287	-2.48	-3.26, -1.69	0.12	92%	110.14	<0.001
Basketball exercises	2	34	34	-1.12	-2.21, -0.02		66	2.94	0.09
Combined exercise	3	97	94	-2.73	-5.18, -0.27		93%	73.25	<0.001

#### 3.4.2 Sensitivity analysis

Sensitivity analysis of the included literature showed that no single study changed the overall outcome.

#### 3.4.3 Publication bias

**Figure 4:**
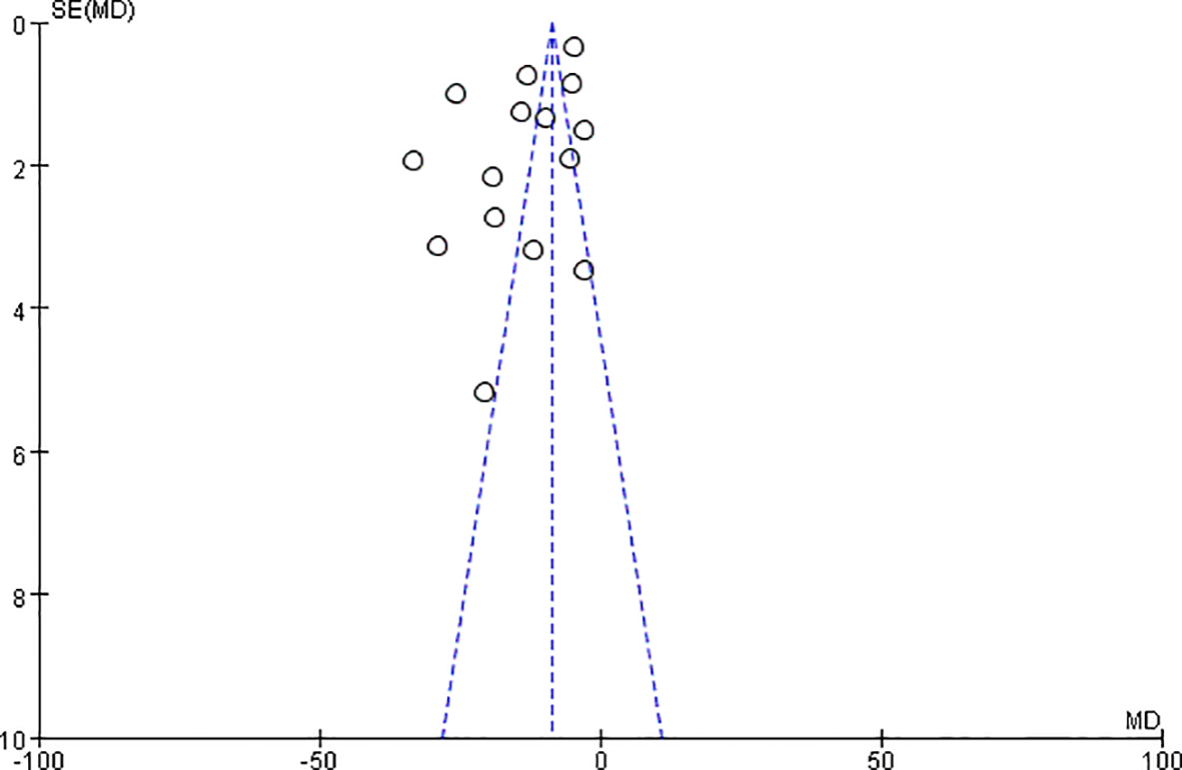
Funnel plot for the publication bias of adolescents or young adults’ IA.

The funnel plot showed potential publication bias (**Figure 4**). The collective results indicated that potential publication bias did not significantly affect the results of this meta-analysis.

### 3.5 Network meta-analysis

To examine the differences in effects among the different interventions, network meta-analyses were further performed.

#### 3.5.1 Network diagram

**Figure 5:**
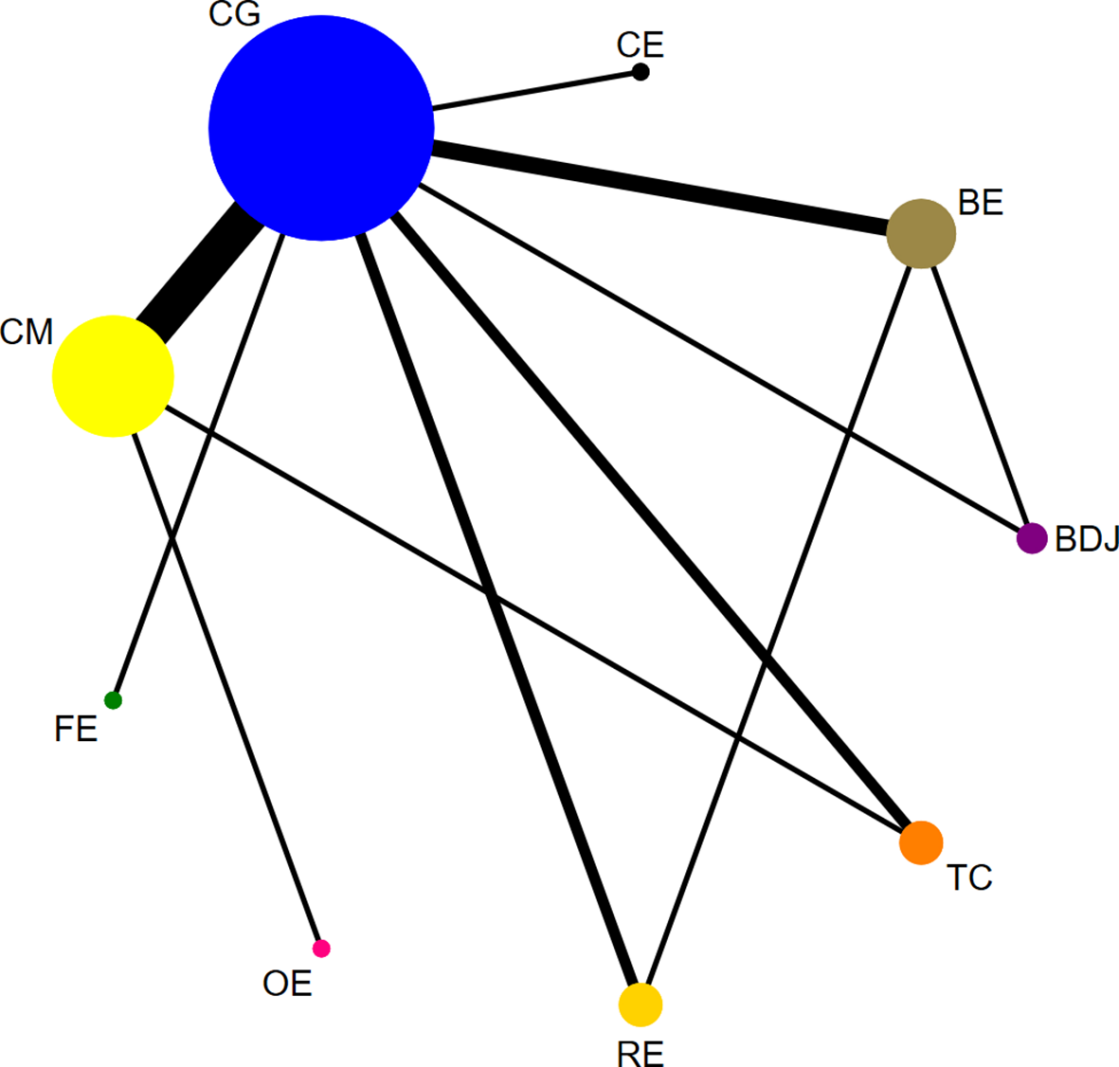
Node Size: The size of each node is now proportional to the total number of participants assigned to that intervention arm. Edge Width: The thickness of the connecting lines (edges) is now proportional to the number of studies contributing to that direct comparison. Color Scheme: A clear color scheme is used to distinguish different types of interventions (CM is yellow, FE is green, OE is pink, RE is orange, BDJ is purple, BE is brown, and CE is black). Network diagram of IA. Tai Chi (TC), Baduanjin (BDJ), Other exercise (OE), Running exercise (RE), Cycling exercise (CE), Combined movement (CM), Basketball exercise (BE), Football exercise (FE), Control group (CG).

As shown in **Figure 5**, the dots in the figure represent the number of subjects in each group; the larger the dots are, the larger the sample size of the subjects. The lines connecting the dots represent the number of original studies directly compared in pairs; the thicker the lines are, the more original studies there are.

#### 3.5.2 Inconsistency of the network

The global inconsistency test revealed p = 0.5567 > 0.05, which can be analyzed for consistency. The local inconsistency test for each closed-loop result using the node-splitting method showed that the p-value of all interventions was >0.05, indicating that the consistency of each closed loop was improved and that the direct and indirect comparisons met the reticulated meta-analysis’s consistency (**Appendix 2**).

#### 3.5.3 Contribution plot

**Figure 6:**
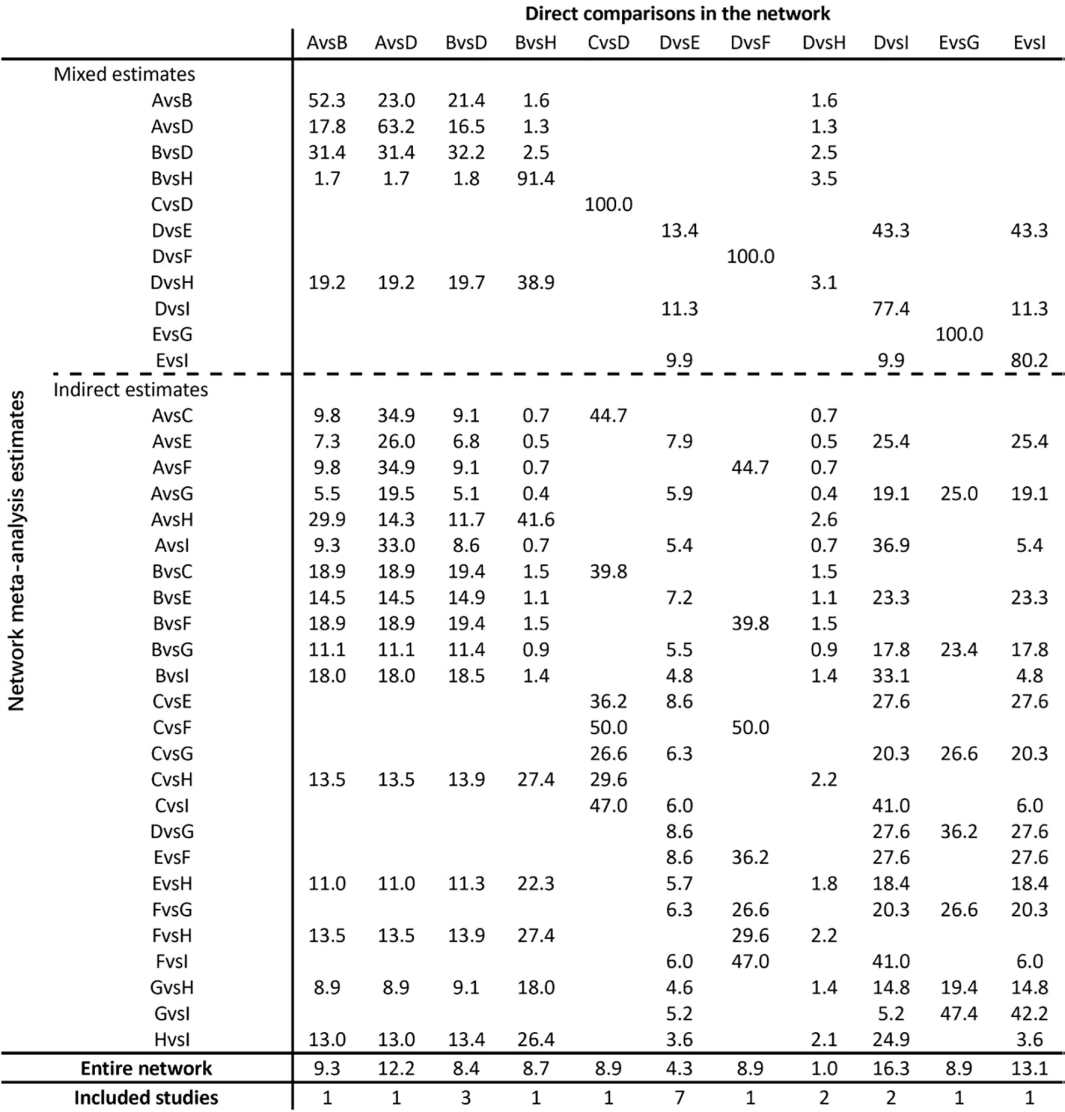
Contribution plot. Baduanjin (BDJ), Basketball exercise (BE), Cycling exercise (CE), Control group (CG), Combined movement (CM), Football exercise (FE), Other exercise (OE), Running exercise (RE), Tai Chi (TC).

The contributions of direct and indirect comparisons to network meta-analysis and the number of studies of each direct comparison are shown in **Figure 6**.

#### 3.5.4 Results of network meta-analysis

**Figure 7:**
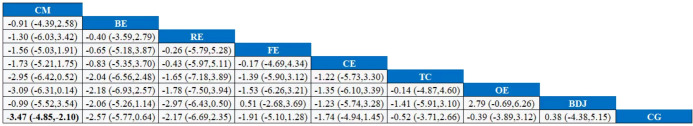
Results of network meta-analysis.

**Figure 8:**
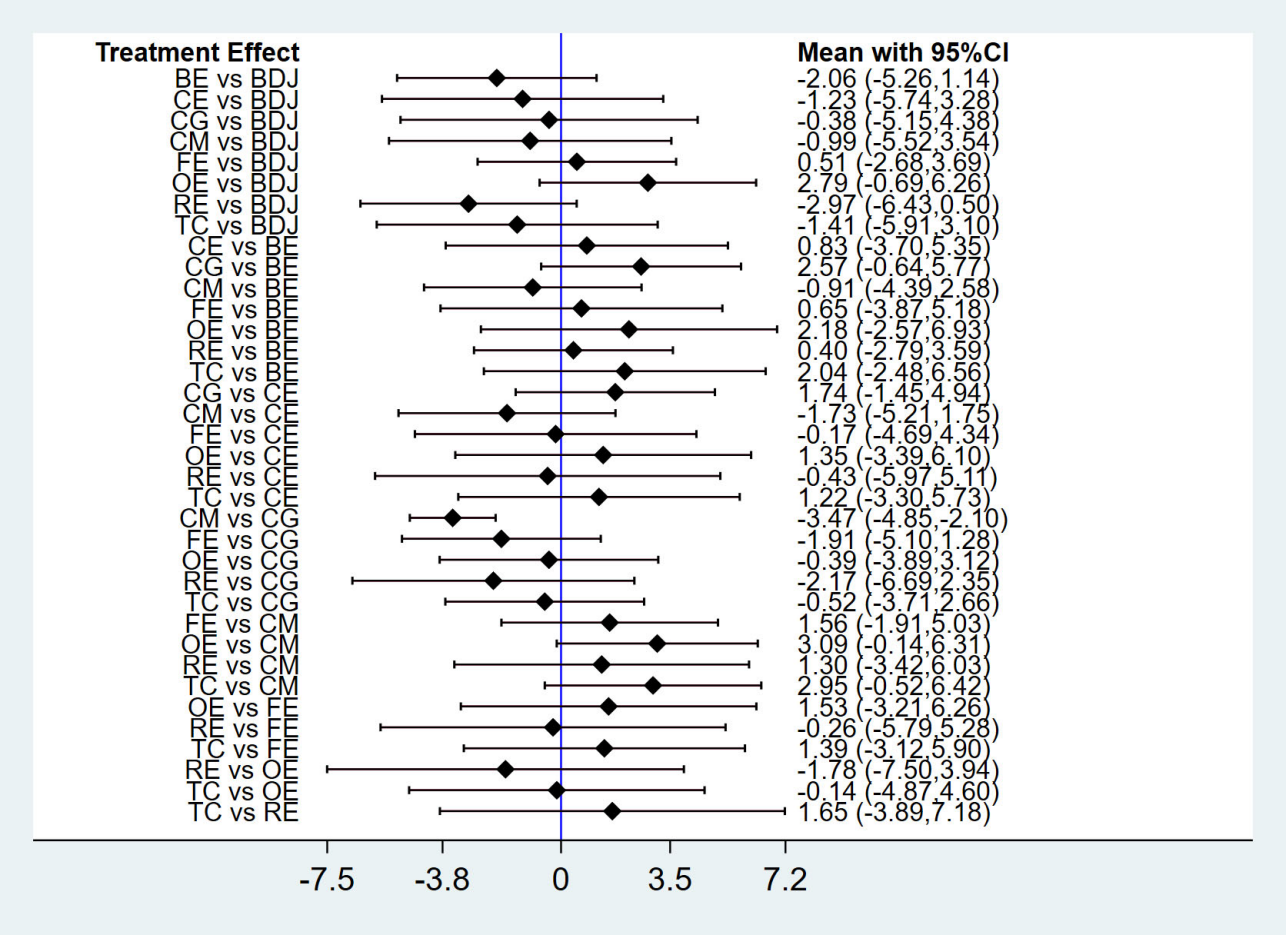
Forest plot for network meta-analysis.

The network meta-analysis showed that CM was significantly better than CG (SMD-3.47, 95% -4.85, -2.10), as shown in **Figure 7**. Forest plots of eligible comparisons are shown in **Figure 8**.

#### 3.5.5 Intervention effect ranking

The SUCRA probability of each intervention in the network is shown in **Appendix 3**. The SUCRA value (**Table 4**) is the probability that each intervention is among the best of those in the network, with larger values representing higher-ranking probabilities.

**Table 4:** The SUCRA values of the interventions.

Treatment	SUCRA	Prbest	Mean rank
Baduanjin (BDJ)	32.3	1.0	6.4
Basketball exercise (BE)	70.5	13.9	3.4
Cycling exercise (CE)	55.7	10.0	4.5
Control group (CG)	20.8	0.0	7.3
Combined movement (CM)	86.7	41.0	2.1
Football exercise (FE)	57.2	11.2	4.4
Other exercise (OE)	32.3	1.7	6.4
Running exercise (RE)	60.2	19.2	4.2
Tai Chi (TC)	34.2	1.9	6.3

#### 3.5.6 Risk of bias across studies

**Figure 9:**
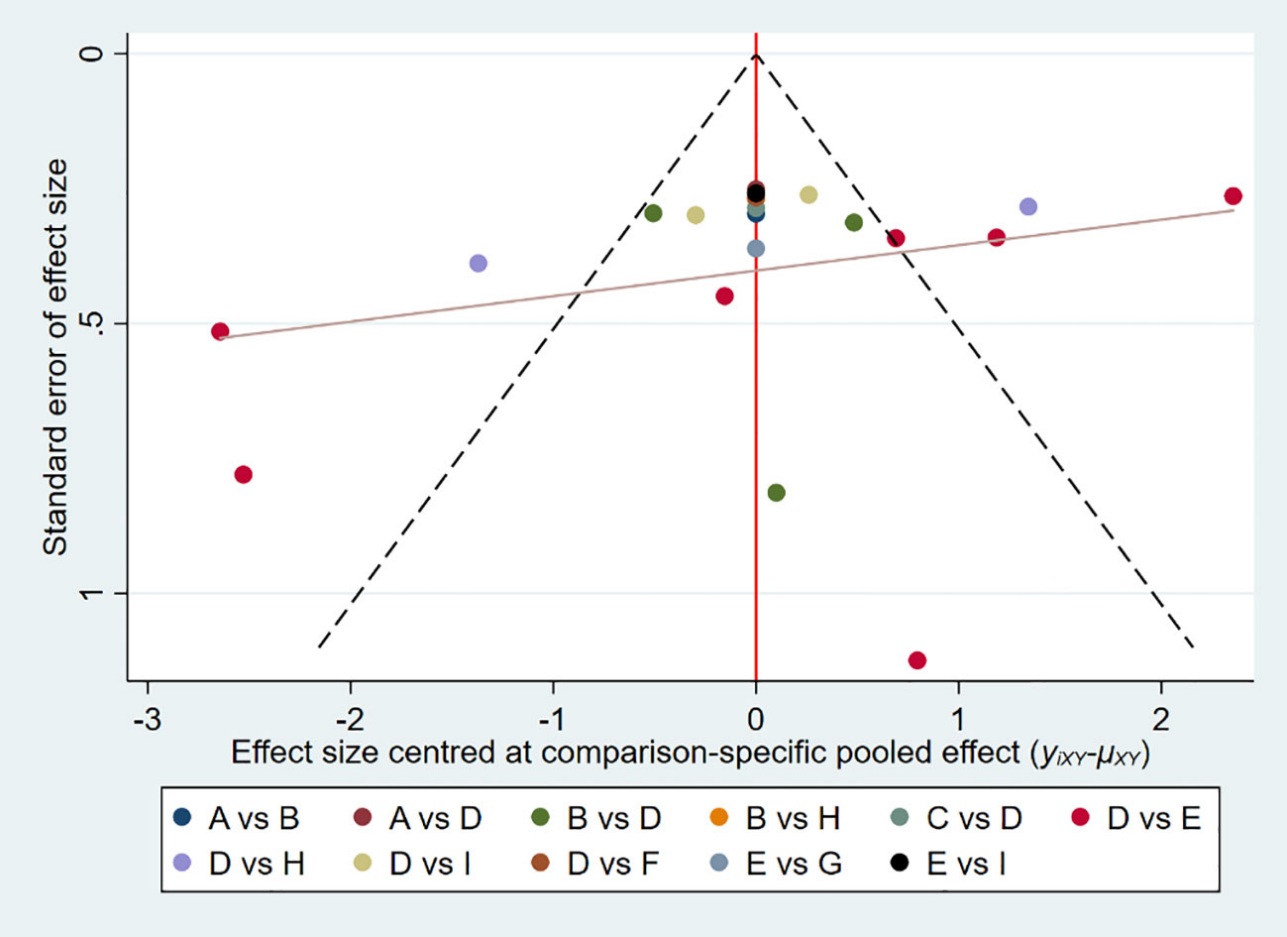
Comparison-adjusted funnel plot of adolescent IA scores.

The publication bias was illustrated by funnel plots (**Figure 9**). According to the network meta-analysis, the funnel plot showed slight asymmetry.

## 4 Discussion

The study included 2013 randomized controlled studies from seven databases. To assess the effects of interventions versus treatment controls, a traditional meta-analysis of 15 studies was performed. In addition, a network meta-analysis of 9 different exercise interventions in 15 studies was conducted to analyze the direct and indirect comparisons between different exercise measures.

### 4.1 Meta-analysis

We conducted a meta-analysis of 15 randomized controlled trials to assess the impact of exercise interventions on IA. The results showed that the exercise intervention had a significant effect on reducing the total score of IA compared with the untreated control group. Total amount effect for [SMD= -2.33, 95%CI (-3.00, -1.66)]. This result is similar to that of Wu (38). Physical activity can enhance regional cerebral blood flow and accelerate metabolic processes, thereby improving the efficiency of cognitive processing in the brain. It facilitates the rapid retrieval of relevant information, reduces external distractions, and supports effective decision-making in adolescents or young adults with IA, ultimately enhancing cognitive function (4). Research on substance dependence has demonstrated that physical exercise can enhance the precise identification of addiction-related stimuli in the initial stages. It helps redirect the attention of individuals with addiction, minimizing cognitive conflict and resource depletion, while also reducing attentional bias towards addiction-related cues (39). In addition, sports can improve the top-down inhibitory control ability of the prefrontal cortex, reduce the psychological craving of people with addiction (40), promote the rational allocation of attention resources in the brain of adolescents or young adults in IA, improve the cognitive processing function in the early stage, and improve the attention bias to addiction-related cues. Furthermore, we performed a subgroup analysis of the findings based on factors such as sample size, assessment tools, intervention duration, intervention frequency, and scale. Given the considerable heterogeneity among the studies, the results should be regarded as preliminary.

### 4.2 Network meta-analysis

On this basis, the network meta-analysis was carried out further to analyze the matched intervention effect of each exercise intervention, and the intervention measures were ranked.

Our results found that CM was superior to the control group in reducing IA among adolescents or young adults. A single way of exercise may make teenagers feel bored and prone to boredom. “Exercise +” combined intervention has become a new trend in the treatment of IA. Compared with a single intervention, combined exercise is more helpful in alleviating the severity and mental symptoms of adolescents or young adults with IA and effectively improves the withdrawal rate of IA (4). The mechanism of action may be that combined intervention has a superposition effect or complementary effect on the treatment of adolescents or young adults with IA. In addition, the diversified types of sports in joint sports can help Internet addicts find the most suitable activities for their different needs. This approach can stimulate interest, reduce monotony, enhance participants’ motivation, and ultimately help reduce IA among adolescents or young adults. Different exercise modalities create varied rewards, thereby preventing tolerance development from repetitive routines (41). Furthermore, studies have found that CM activate the brain’s reward circuitry and directly regulate dopamine signaling (42). This process generates physiological pleasure and a sense of excitement (43), which can, to some extent, substitute for the reward stimulation derived from addictive behaviors. Consequently, the reinforcing effects of internet-based stimuli are weakened, thereby reducing cravings for IA among adolescents and young adults.

### 4.3 Strengths and limitations

As China is the most populous country in the world, IA poses a significant challenge to society and public health. Finding an effective strategy to solve this problem remains an urgent issue. In our research, we further verified that exercise is an effective measure to reduce IA, and through further exploration by NMA, it was discovered CM is the best movement for reducing IA among teenagers. However, our research still has some limitations: all the studies we included were from China, and the inferences drawn from the results should be conducted with caution. Previous studies have shown that the intensity of exercise plays a crucial role in reducing IA among teenagers (44–46). In our study, the reported exercise intensity was either moderate or not reported at all. Furthermore, our research produced an interesting finding: only CM had a statistically significant advantage over CG, while no significant differences were observed in other movement interventions. This might be due to the fact that the number of included studies is relatively small compared with other exercise methods. The ranking of exercise interventions is based on the average SUCRA score, which does not mean that the higher-ranked intervention measures are statistically superior to the lower-ranked ones. Therefore, the research results should be interpreted with caution. Our research classified different types of sports, yet we did not categorize them based on the types of sports interactions. In view of the above limitations, we suggest that in the future, sports interactions can be classified according to their types (for example, team, pair, and individual sports). In the research, more attention should be paid to the intensity of exercise, a wider range of exercise methods should be included, and a more rigorous experimental design should be adopted to gain deeper insights. The included studies in our analysis utilized different measurement instruments. Although we have identified partial sources of heterogeneity through meta-regression and subgroup analyses, variations in assessment tools may still exert a potential influence on the results. We recommend that future research adopt more standardized inclusion criteria and rigorously validated measurement tools to further verify the findings of this study.

## 5 Conclusion

Our findings demonstrate that all exercise interventions examined in this study significantly reduced IA. Based on the network meta-analysis results, combined exercise may be the best intervention. Future research will expand the scope of exercise interventions to incorporate diverse modalities (e.g., aerobics, badminton, combat gymnastics, and diving) to establish an evidence-based framework for optimal intervention selection. However, it is worth noting that the best exercise methods we have identified are based on the results of our statistical analysis. In actual intervention, comprehensive considerations should be made to select the most personalized exercise intervention methods to improve IA among adolescents or young adults.

## Data Availability

The original contributions presented in the study are included in the article/**Supplementary Material**. Further inquiries can be directed to the corresponding author.
