# Supplementary material for: The effects of different exercise interventions on reducing internet addiction in adolescents or young adults: a systematic review and network meta-analysis
Source: Front Psychiatry. 2025 Nov 26;16:1713076. doi: 10.3389/fpsyt.2025.1713076 (PMC12690190; doi:10.3389/fpsyt.2025.1713076)
Supplement: Supplementary file 1 [file DataSheet1.zip › Supplementary Material/Appendix 1-Search strategy.DOCX]

**Pubmed:**

| Number | Search terms | Results |
| --- | --- | --- |
| **#1** | (((((((((((((((((((((((((Internet addiction[MeSH Terms]) OR (Addiction Disorder, Internet[Title/Abstract])) OR (Addiction Disorders, Internet[Title/Abstract])) OR (Disorder, Internet Addiction[Title/Abstract])) OR (Disorders, Internet Addiction[Title/Abstract])) OR (Internet Addiction Disorders[Title/Abstract])) OR (Internet Addiction[Title/Abstract])) OR (Addiction, Internet[Title/Abstract])) OR (Addictions, Internet[Title/Abstract])) OR (Internet Addictions[Title/Abstract])) OR (Internet Gaming Disorder[Title/Abstract])) OR (Disorder, Internet Gaming[Title/Abstract])) OR (Disorders, Internet Gaming[Title/Abstract])) OR (Gaming Disorder, Internet[Title/Abstract])) OR (Gaming Disorders, Internet[Title/Abstract])) OR (Internet Gaming Disorders[Title/Abstract])) OR (Smartphone Addiction[Title/Abstract])) OR (Addiction, Smartphone[Title/Abstract])) OR (Addictions, Smartphone[Title/Abstract])) OR (Smartphone Addictions[Title/Abstract])) OR (Social Media Addiction[Title/Abstract])) OR (Addiction, Social Media[Title/Abstract])) OR (Addictions, Social Media[Title/Abstract])) OR (Media Addiction, Social[Title/Abstract])) OR (Media Addictions, Social[Title/Abstract])) OR (Social Media Addictions[Title/Abstract]) | 7014 |
| **#2** | ((((((((((((((((((((((((((((((Exercises[Title/Abstract]) OR (Exercise, Physical[Title/Abstract])) OR (Exercises, Physical[Title/Abstract])) OR (Physical Exercise[Title/Abstract])) OR (Physical Exercises[Title/Abstract])) OR (Physical Activity[Title/Abstract])) OR (Activities, Physical[Title/Abstract])) OR (Activity, Physical[Title/Abstract])) OR (Physical Activities[Title/Abstract])) OR (Exercise, Aerobic[Title/Abstract])) OR (Aerobic Exercise[Title/Abstract])) OR (Aerobic Exercises[Title/Abstract])) OR (Exercises, Aerobic[Title/Abstract])) OR (Exercise, Isometric[Title/Abstract])) OR (Exercises, Isometric[Title/Abstract])) OR (Isometric Exercises[Title/Abstract])) OR (Isometric Exercise[Title/Abstract])) OR (Acute Exercise[Title/Abstract])) OR (Acute Exercises[Title/Abstract])) OR (Realities, Instructional Virtual[Title/Abstract])) OR (Reality, Instructional Virtual[Title/Abstract])) OR (Virtual Realities, Instructional[Title/Abstract])) OR (Treadmill Training[Title/Abstract])) OR (Resistance Training[Title/Abstract])) OR (Tai Chi[Title/Abstract])) OR (Dance[Title/Abstract])) OR (Game training[Title/Abstract])) OR (Baduanjin[Title/Abstract])) OR (Pilates exercise[Title/Abstract])) OR (Yoga[Title/Abstract])) OR (Aerobic exercise[Title/Abstract]) | 282,123 |
| **#3** | #1 AND #2 | 268 |

**Web of science:**

| Number | Search terms | Results |
| --- | --- | --- |
| **#1** | Title = Internet addiction OR Addiction Disorder, Internet OR Addiction Disorders, Internet OR Disorder, Internet Addiction OR Disorders, Internet Addiction OR Internet Addiction Disorders OR Internet Addiction OR Addiction, Internet OR Addictions, Internet OR Internet Addictions OR Internet Gaming Disorder OR Disorder, Internet Gaming OR Disorders, Internet Gaming OR Gaming Disorder, Internet OR Gaming Disorders, Internet OR Internet Gaming Disorders OR Smartphone Addiction OR Addiction, Smartphone OR Addictions, Smartphone OR Smartphone Addictions OR Social Media Addiction OR Addiction, Social Media OR Addictions, Social Media OR Media Addiction, Social OR Media Addictions, Social OR Social Media Addictions | 5985 |
| **#2** | Title = Exercises OR Physical Exercise OR Physical Exercises OR Physical Activity OR Activities, Physical OR Activity, Physical OR Physical Activities OR Exercise, Aerobic OR Aerobic Exercise OR Aerobic Exercises OR Exercises, Aerobic OR Exercise, Isometric OR Exercises, Isometric OR Isometric Exercises OR Isometric Exercise OR Acute Exercise OR Acute Exercises OR Realities, Instructional Virtual OR Reality, Instructional Virtual OR Virtual Realities, Instructional OR Treadmill Training OR Resistance Training OR Tai Chi OR Dance OR Game training OR Baduanjin OR Pilates exercise OR Yoga OR Aerobic exercise | 344,613 |
| **#3** | #1 AND #2 | 112 |

**Embase:**

| Number | Search terms | Results |
| --- | --- | --- |
| **#1** | 'addiction disorder, internet' OR (('addiction'/exp OR addiction) AND ('disorder,'/exp OR disorder,) AND ('internet'/exp OR internet)) OR 'addiction disorders, internet' OR (('addiction'/exp OR addiction) AND ('disorders,'/exp OR disorders,) AND ('internet'/exp OR internet)) OR 'disorder, internet addiction' OR (('disorder,'/exp OR disorder,) AND ('internet'/exp OR internet) AND ('addiction'/exp OR addiction)) OR 'disorders, internet addiction' OR (('disorders,'/exp OR disorders,) AND ('internet'/exp OR internet) AND ('addiction'/exp OR addiction)) OR 'internet addiction disorders' OR (('internet'/exp OR internet) AND ('addiction'/exp OR addiction) AND ('disorders'/exp OR disorders)) OR 'internet addiction'/exp OR 'internet addiction' OR (('internet'/exp OR internet) AND ('addiction'/exp OR addiction)) OR 'addiction, internet'/exp OR 'addiction, internet' OR (('addiction,'/exp OR addiction,) AND ('internet'/exp OR internet)) OR 'addictions, internet' OR (addictions, AND ('internet'/exp OR internet)) OR 'internet addictionsor internet gaming disorder' OR (addictionsor AND ('internet'/exp OR internet) AND gaming AND ('disorder'/exp OR disorder)) OR 'disorder, internet gaming' OR (('disorder,'/exp OR disorder,) AND ('internet'/exp OR internet) AND gaming) OR 'disorders, internet gaming' OR (('disorders,'/exp OR disorders,) AND ('internet'/exp OR internet) AND gaming) OR 'gaming disorder, internet' OR (gaming AND ('disorder,'/exp OR disorder,) AND ('internet'/exp OR internet)) OR 'gaming disorders, internet' OR (gaming AND ('disorders,'/exp OR disorders,) AND ('internet'/exp OR internet)) OR 'internet gaming disorders' OR (('internet'/exp OR internet) AND gaming AND ('disorders'/exp OR disorders)) OR 'smartphone addictionor addiction, smartphone' OR (addictionor AND ('addiction,'/exp OR addiction,) AND ('smartphone'/exp OR smartphone)) OR 'addictions, smartphone' OR (addictions, AND ('smartphone'/exp OR smartphone)) OR 'smartphone addictions' OR (('smartphone'/exp OR smartphone) AND addictions) OR 'social media addiction'/exp OR 'social media addiction' OR (('social'/exp OR social) AND ('media'/exp OR media) AND ('addiction'/exp OR addiction)) OR 'addiction, social media' OR (('addiction,'/exp OR addiction,) AND ('social'/exp OR social) AND ('media'/exp OR media)) OR 'addictions, social media' OR (addictions, AND ('social'/exp OR social) AND ('media'/exp OR media)) OR 'media addiction, social' OR (('media'/exp OR media) AND ('addiction,'/exp OR addiction,) AND ('social'/exp OR social)) OR 'media addictions, social' OR (('media'/exp OR media) AND addictions, AND ('social'/exp OR social)) OR 'social media addictions' OR (('social'/exp OR social) AND ('media'/exp OR media) AND addictions) | 5985 |
| **#2** | **exercises** OR **'exercise, physical'** OR ((**'exercise,'**/exp OR **exercise,**) AND **physical**) OR **'exercises, physical'** OR (**exercises,** AND **physical**) OR **'physical exercise'**/exp OR **'physical exercise'** OR (**physical** AND (**'exercise'**/exp OR **exercise**)) OR **'physical exercises'** OR (**physical** AND **exercises**) OR **'physical activity'**/exp OR **'physical activity'** OR (**physical** AND (**'activity'**/exp OR **activity**)) OR **'activities, physical'** OR (**activities,** AND **physical**) OR **'activity, physical'**/exp OR **'activity, physical'** OR (**activity,** AND **physical**) OR **'physical activities'** OR (**physical** AND **activities**) OR **'exercise, aerobicor aerobic exercise'** OR ((**'exercise,'**/exp OR **exercise,**) AND **aerobicor** AND **aerobic** AND (**'exercise'**/exp OR **exercise**)) OR **'aerobic exercises'** OR (**aerobic** AND **exercises**) OR **'exercises, aerobic'** OR (**exercises,** AND **aerobic**) OR **'exercise, isometric'**/exp OR **'exercise, isometric'** OR ((**'exercise,'**/exp OR **exercise,**) AND **isometric**) OR **'exercises, isometric'** OR (**exercises,** AND **isometric**) OR **'isometric exercises'** OR (**isometric** AND **exercises**) OR **'isometric exercise'**/exp OR **'isometric exercise'** OR (**isometric** AND (**'exercise'**/exp OR **exercise**)) OR **'acute exercise'**/exp OR **'acute exercise'** OR (**acute** AND (**'exercise'**/exp OR **exercise**)) OR **'acute exercises'** OR (**acute** AND **exercises**) OR **'realities, instructional virtual'** OR (**realities,** AND **instructional** AND **virtual**) OR **'reality, instructional virtual'** OR (**reality,** AND **instructional** AND **virtual**) OR **'virtual realities, instructional'** OR (**virtual** AND **realities,** AND **instructional**) OR **'treadmill training'** OR ((**'treadmill'**/exp OR **treadmill**) AND (**'training'**/exp OR **training**)) OR **'resistance training'**/exp OR **'resistance training'** OR ((**'resistance'**/exp OR **resistance**) AND (**'training'**/exp OR **training**)) OR **'tai chi'**/exp OR **'tai chi'** OR (**tai** AND **chi**) OR **'dance'**/exp OR **dance** OR **'game training'** OR ((**'game'**/exp OR **game**) AND (**'training'**/exp OR **training**)) OR **'baduanjin'**/exp OR **baduanjin** OR **'pilates exercise'**/exp OR **'pilates exercise'** OR ((**'pilates'**/exp OR **pilates**) AND (**'exercise'**/exp OR **exercise**)) OR **'yoga'**/exp OR **yoga** OR **'aerobic exercis'** OR (**aerobic** AND **exercis**) | 1,556,101 |
| **#3** | #1 AND #2 | 1005 |

**Cochrane:**

| Number | Search terms | Results |
| --- | --- | --- |
| **#1** | Internet addiction OR Addiction Disorder, Internet OR Addiction Disorders, Internet OR Disorder, Internet Addiction OR Disorders, Internet Addiction OR Internet Addiction Disorders OR Internet Addiction OR Addiction, Internet OR Addictions, Internet OR Internet Addictions OR Internet Gaming Disorder OR Disorder, Internet Gaming OR Disorders, Internet Gaming OR Gaming Disorder, Internet OR Gaming Disorders, Internet OR Internet Gaming Disorders OR Smartphone Addiction OR Addiction, Smartphone OR Addictions, Smartphone OR Smartphone Addictions OR Social Media Addiction OR Addiction, Social Media OR Addictions, Social Media OR Media Addiction, Social OR Media Addictions, Social OR Social Media Addictions | 1287 |
| **#2** | Physical OR Physical Activities OR Exercise, Aerobic OR Aerobic Exercise OR Aerobic Exercises OR Exercises, Aerobic OR Exercise, Isometric OR Exercises, Isometric OR Isometric Exercises OR Isometric Exercise OR Acute Exercise OR Acute Exercises OR Realities, Instructional Virtual OR Reality, Instructional Virtual OR Virtual Realities, Instructional OR Treadmill Training OR Resistance Training OR Tai Chi OR Dance OR Game training OR Baduanjin OR Pilates exercise OR Yoga OR Aerobic exercise | 171,470 |
| **#3** | #1 AND #2 | 240 |

**CNKI:**

| Number | Search terms | Results |
| --- | --- | --- |
| **#1** | 主题= 网络成瘾 + 网瘾 + 网络沉迷 + 互联网成瘾 | 12,757 |
| **#2** | 主题= 练习 + 锻炼 + 体育 + 锻炼 + 体育活动 + 健美操 + 有氧运动 + 急性运动 + 八段锦 + 太极拳 + 太极 + 瑜伽 + 篮球 + 足球 + 排球 + 球类 + 跑步 | 1,391,379 |
| **#3** | #1 AND #2 | 119 |

**VIP Database :**

| Number | Search terms | Results |
| --- | --- | --- |
| **#1** | 题目与关键词= 网络成瘾 + 网瘾 + 网络沉迷 + 互联网成瘾 | 12,757 |
| **#2** | 题目与关键词= 练习 + 锻炼 + 体育 + 锻炼 + 体育活动 + 健美操 + 有氧运动 + 急性运动 + 八段锦 + 太极拳 + 太极 + 瑜伽 + 篮球 + 足球 + 排球 + 球类 + 跑步 | 7258 |
| **#3** | #1 AND #2 | 165 |

**VIP Database :**

| Number | Search terms | Results |
| --- | --- | --- |
| **#1** | 题目与关键词= 网络成瘾 + 网瘾 + 网络沉迷 + 互联网成瘾 | 14,398 |
| **#2** | 题目与关键词= 练习 + 锻炼 + 体育 + 锻炼 + 体育活动 + 健美操 + 有氧运动 + 急性运动 + 八段锦 + 太极拳 + 太极 + 瑜伽 + 篮球 + 足球 + 排球 + 球类 + 跑步 | 2,292,870 |
| **#3** | #1 AND #2 | 104 |
