# Supplementary material for: The effects of different exercise interventions on reducing internet addiction in adolescents or young adults: a systematic review and network meta-analysis
Source: Front Psychiatry. 2025 Nov 26;16:1713076. doi: 10.3389/fpsyt.2025.1713076 (PMC12690190; doi:10.3389/fpsyt.2025.1713076)
Supplement: Supplementary file 1 [file DataSheet1.zip › Supplementary Material/Appendix 3- meta-regression.docx]

**Appendix 3- meta-regression**

|  | Coef. | Std. Err. | t | P>\|t\| |
| --- | --- | --- | --- | --- |
| **Outcome measurement** | 1.877 | 0.190 | 9.89 | ＜0.001 |
| **Intervention frequency** | 2.409 | 0.201 | 11.97 | ＜0.001 |
| **Intervention mode** | 1.501 | 0.183 | 8.18 | ＜0.001 |
| **Year of publication** | 1.379 | 0.140 | 9.87 | ＜0.001 |
| **Sample size** | 1.085 | 0.090 | 12.09 | ＜0.001 |
| **Intervention duration** | 2.003 | 0.211 | 9.52 | ＜0.001 |
