# Supplementary figures and images for: The effects of different exercise interventions on reducing internet addiction in adolescents or young adults: a systematic review and network meta-analysis
Source: Front Psychiatry. 2025 Nov 26;16:1713076. doi: 10.3389/fpsyt.2025.1713076 (PMC12690190; doi:10.3389/fpsyt.2025.1713076)

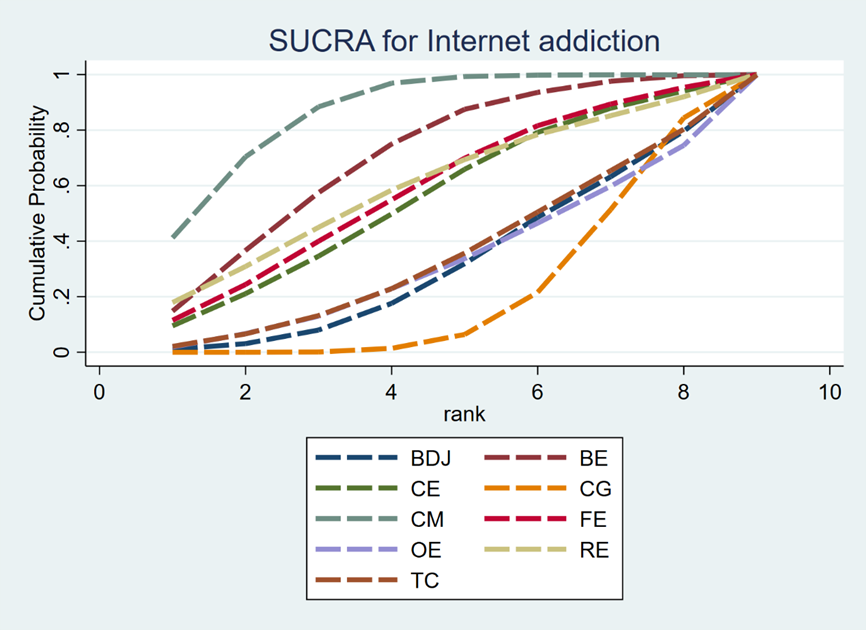


**Fig. 9. Sucra graph of effectiveness among interventions.**

Supplement: Supplementary file 1 [file DataSheet1.zip › Supplementary Material/Appendix 4- Sucra graph.docx]
